# Biomaterial Influence on Intraocular Lens Performance: An Overview

**DOI:** 10.1155/2018/2687385

**Published:** 2018-03-15

**Authors:** Cari Pérez-Vives

**Affiliations:** Alcon Management SA, Geneva, Switzerland

## Abstract

There is strong evidence that the IOL material is the factor having the greatest impact on posterior capsule opacification (PCO), anterior capsule opacification (ACO) development, and glistening formation after cataract surgery, even though there are other IOL features—such as haptic material and design and edge and optic design—that also have some influence. We reviewed the published literature describing the adverse events that are mainly related to the intraocular lens (IOL) material, such as PCO, ACO, and the subsequent capsule contraction, as well as glistening formation. The adverse events presented in this overview are the most common ones in clinical practice, and therefore, they are generally included in the clinical protocols for IOL evaluation.

## 1. Introduction

Cataract is at present the second cause of blindness worldwide after age-related macular degeneration; moreover, in Eastern and Central Europe, cataract is still the leading cause [1]. In the United States (US) alone, cataract affects—in at least one eye—an estimated 20.5 million people (17.2%) over 40 years of age, and 6.1 million (5.1%) people have pseudophakia/aphakia. The total number of US people having cataract is predicted to increase up to 30.1 million by 2020, and 9.5 million of them are expected to have pseudophakia/aphakia [2]. Consequently, cataract surgery is one of the most frequent surgical procedures.

Cataract surgery is constantly evolving and improving in terms of the lens material and designs. The first intraocular lenses (IOLs), which were manufactured in 1949, were made of rigid plastic—namely, polymethyl methacrylate (PMMA)—and this biomaterial was the only one available for IOL implantation for over 30 years. The first implanted PMMA IOLs were implanted through an extracapsular surgical technique that resulted in large incisions and induced postoperative astigmatism. Subsequently, the design and the surgical technique have greatly improved. In the early 1970s, Charles Kelman introduced phacoemulsification, thus reducing the incision size, and initiated biomaterial diversification, including foldable materials such as silicone, hydrogel, and acrylic compounds [3, 4]. Today, cataract surgery is mainly performed using phacoemulsification and a foldable IOL, which is implanted through a small incision.

There are many types of IOLs, in terms of both optic features and materials. IOL design and materials are constantly evolving fields, aiming for better refractive outcomes with minimal incision size and trying to minimize host-cell response, since it may cause posterior capsule opacification (PCO), anterior capsular opacification (ACO), and lens epithelial cell (LEC) proliferation. IOL materials vary in water content, chemical composition, refractive index, and tensile strength, while IOL designs have different optic size, edge profiles, and haptic materials and designs, with the main goal of minimizing decentration, dislocations, optical aberrations, and opacifications [5–7].

The literature search was conducted in the Embase®, Medline® and Medline In-process, and the Cochrane databases through Embase and Ovid® platforms from January 2000 to December 2015. A detailed search strategy using terms related to cataract, intraocular lens, posterior capsular opacification, Nd:YAG laser, anterior capsule opacification, capsule contraction, and glistenings was prepared, to identify published literature reporting evidence that matched our research objectives.

This review provides an overview of the currently available IOL materials and designs and discusses their effect upon PCO, ACO, capsule contraction, and glistening formation.

## 2. Posterior Capsule Opacification

Surgical trauma during the surgery initially causes blood-aqueous barrier breakdown. This leads to protein leakage and macrophage migration from the blood into the surgical zone, eliciting immediate postoperative inflammation. The cellular reaction consists of two main components. One component comprises large cells, macrophages, and epithelioid cells, which later coalesce to form foreign body type giant cells, which represent uveal biocompatibility. The other comprises LECs, which can be divided into PCO and ACO. The source of the PCO is the equatorial cells originating from the equator of the capsule. These cells go through metaplasia and acquire the ability to migrate and proliferate, causing epithelial ingrowth between the IOL and the posterior capsule, which leads to a reduction in VA. PCO still remains the most frequent complication of modern cataract surgery. Advances in surgical techniques and IOL materials and designs have reduced PCO rates, but it is still a significant problem in clinical practice [8]. PCO can be effectively treated by using a neodymium:YAG (Nd:YAG) laser to cut a hole in the posterior lens capsule. However, this procedure may lead to additional complications, including IOL damage, intraocular pressure (IOP) elevation, glaucoma, cystoid macular edema, or even retinal detachment [9]. The pathophysiology of PCO is multifactorial, varying particularly with the surgical technique, IOL materials, and designs [10–14], but since dissociation of each factor involved in PCO development is almost impossible, it is very difficult to differentiate between the individual elements in clinical practice.

Regarding the IOL material, the Linnola “sandwich theory” states that bioactive materials allow a single LEC to bond to both the IOL and the posterior capsule. This produces a sandwich pattern including the IOL, the LEC monolayer, and the posterior capsule, thus preventing further cell proliferation and capsular bag opacification [15]. Other studies carried out by Linnola et al. evaluated the adhesiveness of fibronectin, vitronectin, laminin, and type-IV collagen to IOL materials (PMMA, silicone, hydrophobic acrylate, and hydrogel), both *in vitro* [16] and in cadaver eyes [17, 18]. They found that fibronectin and laminin bond best to hydrophobic acrylate IOLs, resulting in better attachment to the capsule. This stronger binding could explain the enhanced adhesion of hydrophobic acrylate IOL to the anterior and posterior capsules and, as a result, the lower PCO and Nd:YAG capsulotomy rates [14, 19–34]. However, observational studies in animals [35] and cadaver eyes [36] showed that the IOL having a sharp posterior optic edge may play a more relevant role in this effect than the IOL material.

Table 1 shows the PCO and Nd:YAG rates reported in previous studies carried out for different IOL materials and designs [12–14, 19–34, 37–47]. Hydrophilic acrylic (including those hydrophilic IOLs with the hydrophobic surface) [25] and PMMA IOLs are associated with higher PCO rates, greater PCO severity, and also higher Nd:YAG capsulotomy rates than hydrophobic acrylic IOLs. Several comparative studies showed a superior reduction in PCO and laser capsulotomy rates with hydrophobic acrylic IOLs, compared with hydrophilic acrylic ones.

Auffarth et al. [20] analyzed PCO and Nd:YAG laser treatment as a function of the IOL material (PMMA, silicone, hydrophilic acrylic, and hydrophobic acrylic). After 3 years of follow-up, hydrophobic acrylic IOLs showed a statistically lower incidence of PCO and Nd:YAG rates than the other three materials. A prospective randomized contralateral study [26] reported significantly higher PCO rates in eyes implanted with a hydrophilic hydrogel IOL (Hydroview H60M, B&L) than in eyes having a hydrophobic acrylic IOL (Acrysof MA60BM, Alcon). Moreover, the results revealed that this PCO led to a notably impaired visual acuity. After two years of follow-up, 28% of the eyes in the hydrophilic group and 2% in the hydrophobic acrylic group had required Nd:YAG capsulotomy. Boureau et al., in a retrospective study [23], compared the incidence of Nd:YAG laser treatment for three square-edge IOL models having different design and material compositions. After 3 years of follow-up, the proportion of patients requiring Nd:YAG laser treatment amounted to 12% for the SA60AT (Alcon) group, 25.2% for the AR40e (AMO), and 51% for the XL-Stabi (Zeiss). Gauthier et al. [25] reported 8.8% and 37.2% Nd:YAG rates after 2 years of bilateral implantation of AcrySof ReSTOR (Alcon) and Acri.LISA (Zeiss) multifocal IOLs, respectively. Similarly, Bourdiol Ducasse et al. [22] reported that eyes with AcrySof IOL implants required significantly fewer Nd:YAG laser capsulotomies than those implanted with a Hoya or Akreos IOLs and, therefore, were less prone to Nd:YAG laser treatment complications, thus ensuring better vision at the lowest cost.

All these outcomes could be attributed to the fact that hydrophobic IOLs are capable of adhering to collagen membranes [48], leading to a tighter IOL apposition in the posterior capsular bag and a better adhesiveness—through fibronectin—than other materials [49]. This may result in less space left between the IOL and the posterior capsule for the LECs to migrate. Furthermore, it has been reported that IOLs with hydrophilic surfaces promote proliferation and migration of LECs from the equatorial area to the visual region [50].

The literature shows more controversial results when comparing hydrophobic acrylic versus silicone materials. Several studies [41, 42, 44, 45, 47] have compared PCO and Nd:YAG rates for 3-piece, sharp-edge silicone IOLs (CeeOn 911A, AMO) and 3-piece, sharp-edge hydrophobic acrylic IOLs (AcrySof, Alcon). After 3 years of follow-up, the results showed low PCO and Nd:YAG capsulotomy incidence rates for both IOL groups with no statistically significant differences between them, thus concluding that the sharp-edge foldable IOLs play an important role in preventing PCO irrespective of the material the IOL is made of [41, 44, 47]. However, Vock et al. [45] found significantly higher PCO scores and Nd:YAG capsulotomy rates with hydrophobic acrylic IOLs than with silicone IOLs after 6 years of follow-up. They concluded that aside from the IOL material, differences in the haptic design and the degree of the optic edge sharpness may play a role, and they highlighted the need for longer follow-up studies. In a retrospective and contralateral study, Abhilakh Missier et al. [19] assessed PCO rates in fellow eyes: each patient received one plate-haptic silicone IOL (AA4203VF, Staar) in one eye and a hydrophobic acrylate IOL (AcrySof MA30BA/MA60BM, Alcon) in the contralateral one. After 3 years of follow-up, they found significantly less PCO and lower Nd:YAG laser capsulotomy rates in the eyes implanted with the hydrophobic IOL than in the plate-haptic silicone IOL group. Other studies [12, 21, 39, 43, 45] also compared PCO and Nd:YAG rates resulting from 3-piece, round optic-edge silicone IOL (SI30N/SI40NB, AMO) and 3-piece, sharp-edge hydrophobic acrylic IOL (AcrySof, Alcon) implantation. Most of these studies revealed that following phacoemulsification, PCO rates increased over the years for all groups and that the benefit yielded by the acrylic IOL in terms of showing lower PCO rates faded as the years went by: after a long follow-up period, up to 12 years of follow-up, the PCO and Nd:YAG treatment rates were similar for both groups. These outcomes could be due to the silicone IOL efficiently inhibiting PCO over a long time or to the efficiency drop or loss of the hydrophobic acrylic IOL's sharp edge due to late LEC proliferation, leading to an emerging Soemmering ring in the peripheral capsular bag, which, in its turn, resulted in a reduced or nonexistent PCO preventive effect 4-5 years postoperatively [12].

Regardless of the IOL material, the importance of IOL edge design is widely accepted, and there is considerable agreement among medical communities in favor of a square posterior optic edge for reducing PCO rates and Nd:YAG procedure needs. A systematic review found significantly lower PCO scores for sharp-edge IOLs compared to round-edge models, although there were no clear differences between IOL materials [10]. A square edge on the posterior IOL surface provides a barrier to LEC migration by inducing a capsular bend in the area where it is in contact with the IOL edge [33, 51–54]. Several trials showed no differences in PCO prevention between IOL models that had sharp posterior optic edges—irrespective of IOL material composition or square-edge design—thus indicating that a sharp posterior optic edge is the main factor preventing PCO [13, 41, 55, 56]. Edge sharpness can vary across IOL models. A prospective, single-site, fellow-eye comparative study of two hydrophobic IOLs found higher PCO rates and poorer visual acuity in eyes implanted with the AF-1 YA-60BB IOL (Hoya) versus the AcrySof SN60AT IOL (Alcon) over 24 months of follow-up. The authors attributed these differences to the fact that the AcrySof IOL has a sharper posterior edge profile than the Hoya IOL [13]. Moreover, in a recent 36-month follow-up study [14], the Hoya iMics1 NY-60 IOL showed higher PCO and Nd:YAG capsulotomy rates than AcrySof SN60WF IOL despite the novel Hoya iMics1 NY 60 model with optimized sharp edges, which are even sharper than those of the AcrySof SN60WF IOL (Alcon). These results suggest that the IOL material continues to play an important role in this complication [14].

A meta-analysis performed by Cheng et al. [11] concluded that PCO and Nd:YAG laser capsulotomy rates may be influenced by two parameters: IOL material and optic edge design. Lenses made of acrylic and silicone and those having sharp optic edges lead to lower PCO and laser capsulotomy rates. Morgan-Warren and Smith [32], in a retrospective, comparative 2-year follow-up study, reported lower Nd:YAG procedure rates with the newer sharper Hoya PY60AD IOL than with the Hoya FY60AD IOL model. The one-piece AcrySof SN60WF IOL was also analyzed in this retrospective study, showing the lower Nd:YAG rates than both the three-piece Hoya IOLs in the same period postoperatively. The sharpness of the square edge of the AcrySof IOL lies between the two Hoya models; therefore, the authors concluded that the IOL edge may contribute to reducing the PCO rates, but variations in IOL material composition may influence IOL's susceptibility to PCO development independent of edge sharpness.

The fact that the primary site of late intrusion of LECs is inside the haptic loop [45] reveals the importance of the haptic configuration for PCO formation, such as broad optic-haptic junction [57–59] and haptics angulation [45]. It has been shown that either less angulation, a better haptic memory, a more c-loop shape of the haptic, and/or a thin and perpendicular insertion of the haptic into the optic, or a combination of these seems to result in a more prolonged and permanent barrier effect against late LEC migration [45]. Nixon and Woodcock [60] compared two 1-piece hydrophobic acrylic IOLs; the authors identified Tecnis AAB00 (AMO) as a continuous optic edge and an AcrySof SA60AT (Alcon) as an interrupted optic edge. Their findings, after 2 years of follow-up, showed that those eyes implanted with a continuous 360-degree square-edge IOL had significantly less PCO than those eyes having a square edge with an interrupted optic-haptic junction IOL. These characteristics—360-degree square-edge, angled haptics, increased optic-haptic space, and increased resistance to compression—help position the IOL against the posterior capsule and promote complete circumferential shrink wrapping of the IOL by the capsule. Nonetheless, recent studies [61–63] have shown that after 1 and 3 years of follow-up, the levels of PCO were low for those eyes implanted with an IOL having a continuous sharp and square optic edge (Tecnis ZCB00, AMO) and for those implanted with an IOL having an interrupted square optic edge (AcrySof SA60AT, Alcon) with no statistically significant differences between the two IOL models. However, they did find higher ACO rates in those eyes implanted with the interrupted optic edge IOL.

On the other hand, studies have consistently failed to reach a consensus on the relative merits of one-piece versus three-piece IOLs per se in protecting against PCO development. Some authors reported more PCO [64] and higher Nd:YAG rates [65] in eyes implanted with a one-piece IOLs compared to three-piece models. The thin haptics of the three-piece IOL is believed to allow for better adhesion between the anterior and posterior capsules and bend formation, compared to the bulky haptics of the one-piece models, which enable enhanced posterior LEC migration. However, other studies showed no significant differences in terms of PCO and Nd:YAG rates between one- and three-piece IOLs [58, 65–70].

There are other features that also exert influence upon PCO formation, such as the presence of aspheric surfaces [71], and optic size [72]. Several studies [73, 74] reported decreased PCO rates when the capsulorhexis is in complete contact with the anterior IOL surface. Meacock et al. [72] reported that larger optic IOLs have a lower PCO; this can be explained by the fact that it is easier to get the capsulorhexis on the IOL with a larger optics. On the other side, the LECs may have an increased ability to migrate from the anterior capsule to the posterior capsule when the anterior capsulorhexis is decentered and the lens is tilted off the posterior capsule [73, 74].

## 3. Anterior Capsule Opacification

Unlike the equatorial cells, the anterior cells go through fibrous metaplasia but lack the ability to migrate and proliferate and therefore do not appear outside the margins of the capsulorhexis. Capsule opacification may occur on either the anterior or the posterior capsule and is caused by LECs that remain in the evacuated capsular bag [75]. The source of ACO is the anterior epithelial cells, which originate from beneath the anterior lens capsule. These cells can be classified into 2 subpopulations with distinct properties: The equatorial LEC population resides in the equatorial region of the capsular bag, and following capsular bag evacuation, these cells tend to migrate onto the posterior capsule and proliferate. Meanwhile, the anterior LEC population resides on the anterior capsule leaf and has the potential to undergo myofibroblastic transdifferentiation. While PCO has been regeneratory or fibrotic or both, ACO can only be fibrotic. ACO generally occurs at a much earlier stage in comparison to PCO: sometimes, it develops within one month postoperatively [76]. It has been shown that the area of the anterior capsule opening seems to gradually decrease for up to 6 months postoperatively [76, 77].

The process of opacification of the anterior capsule may be split into four stages: (1) fibrosis/opacification of the capsulorhexis margin in some places; (2) the entire anterior capsular edge in contact with the IOL optic's biomaterial; (3) formation of capsular folds; and (4) advanced/excessive and/or asymmetric shrinkage. Such shrinkage may result in some complications, such as eccentric displacement of the continuous curvilinear capsulorhexis (CCC) opening, IOL decentration [78], capsulorhexis phimosis, and capsule contraction [79]. Some authors recommend Nd:YAG laser anterior relaxing incisions in the early postoperative period after cataract surgery in order to prevent capsule contraction in high-risk patients (such as those with primary angle closure, pseudoexfoliation, or diabetic retinopathy) [80, 81].

There are different factors that could influence the level of ACO, such as CCC's initial size; IOL material and design; and some preexisting conditions (e.g., the quality of the zonular support). Some authors reported that if the CCC is smaller than the diameter of the IOL optic, the contact of the optic's biomaterial with the anterior capsule will induce fibrosis/opacification [82, 83]. Tsuboi et al. [84] studied the influence of the CCC and IOL fixation on the blood-aqueous barrier (BAB). They found an unfavorable effect of in-the-bag fixation with a small CCC and thus a broader contact of the IOL optic with the anterior capsule. On the contrary, Gonvers et al. [85] did not find any correlation between the initial CCC size and the postoperative CCC constriction.

Park et al. [86] evaluated the rate at which the area of the anterior capsule opening decreased following CCC for different IOL types. Although this area reduction occurred for all patients, on average, it was significantly lower in the two acrylic-IOL groups than in the silicone-IOL one. They suggested that IOL selection can be important when it comes to reducing anterior capsule opening contraction, especially for patients at risk for contraction. In this sense, the IOL material and design also play a role in ACO, which is a complication that can impair visual function [87]. Werner et al. [88, 89] evaluated the degree of ACO in postmortem human eyes that had been implanted with IOLs of a wide variety of materials and designs. The results of this histopathological study revealed that ACO is more likely to occur with plate-haptic silicone and hydrogel IOLs than with acrylic hydrophobic IOLs. Nagata et al. [90] also reported a marked anterior capsule contraction with a silicone IOL (AQ310NV, Canon) compared to a hydrophobic acrylic IOL, as well as varying rates of capsule contraction across the range of acrylic IOLs under test (AR40e, AMO; AcrySof MA60BM and SA60AT models, Alcon; and YA60BBR, Hoya), being statistically greater with the AR40e. These results suggest that when implanting an IOL with high surface adhesion, a sharp bend is created in the lens capsule at the rectangular, sharp posterior optic edge of the IOL soon after surgery. This suppresses PCO formation and anterior capsule contraction [78, 91]. In a retrospective study, Tsinopoulos et al. [92] reported significantly greater capsule contraction with hydrophilic IOLs (Quatrix and ACR6D, Corneal Laboratories) than with hydrophobic ones (AcrySof SN60AT, Alcon).

K. Hayashi and H. Hayashi [93] reported that the optic material is the most important factor influencing the degree of capsule contraction, whereas the optic design and haptic material and design play a less significant role. Other studies have shown that plate haptics [85], single-piece designs [94], or IOLs that have a thin optics are risk factors for capsule contraction [95]. Among the four silicone IOL groups assessed by Werner et al. [89], plate-haptic silicone ones yielded significantly higher scores than the 3-piece designs. This correlates well with the findings by Gonvers et al. [85] who claimed that the IOL haptic design (loop-haptic versus plate-haptic) has a major effect on the CCCs' size change. The excessive CCC constriction observed with plate-haptic IOLs is probably due to the relatively large contact area between the plate haptic silicone material and the anterior capsule, in sharp contrast to three-piece IOLs, with which the contact is limited to the optic's surface. Thus, the larger surface exposure inherent to plate IOLs may stimulate cell proliferation and fibrosis. Despite these findings, Sacu et al. [96] showed in a recent study that neither the optic material (silicon versus hydrophobic IOLs) nor the haptic design (one-piece versus three-piece open loop) had any influence on the amount of ACO or capsulorhexis contraction.

Several studies reported that capsule contraction has also been associated with some systemic or ocular conditions, such as diabetes mellitus [97–99], pseudoexfoliation syndrome [100], retinitis pigmentosa [101], and uveitis [79]. It may result in a smaller capsulotomy opening, opening malposition, reduction in equatorial capsular diameter, and displacement of the IOL, this effect being more acute for small capsulorhexis openings and older patient [79]. The Nd:YAG laser can be used to perform anterior capsulotomy to increase the size of the anterior capsule opening, but this treatment can lead to other complications, such as IOP elevation, iritis, corneal edema, and damage to the IOL [9].

## 4. Glistenings

Glistenings are condensations of water within the IOL polymer matrix that occur when the IOL is in an aqueous environment. They are usually distributed throughout the entire IOL optic. They result from the formation of water vacuoles within the lens due to in-the-eye hydration; they are not caused by the deterioration of the material. The mechanism of glistening formation has been extensively evaluated [102]. The polymers that make up the IOLs have different components, including different monomers, chromophores, and crosslinking agents. Polymers absorb water when immersed in an aqueous environment, and their water absorption rate depends on the specific IOL material and the temperature. If the IOL is placed in warm water and the temperature is then lowered, the water inside the polymer becomes oversaturated [103, 104] and, consequently, it separates into phases and collects in a void, generating glistening [105]. In the clinical practice, glistenings typically begin to appear over a 1- to 16-month period after implantation.

Kato et al. [102] found an association between a reduction in temperature and the rate of glistening formation. They studied the changes undergone by a Wagon Wheel packaged AcrySof IOL at various temperatures, placing the IOLs in 37°C or 70°C water, which was then cooled down to 23°C. Microvacuoles of 1.0 to 20.0 *μ*m diameter formed inside the AcrySof IOL when the temperature dropped from 37° to 34°C (3°C decrease), which was enough to trigger vacuole formation. Vacuole density was higher in the IOL that had undergone cooling from 60°C than in the one cooled from only 37°C. In the same paper, the authors also studied temperature changes on the human ocular surface. The temperature of the ocular surface decreased by approximately 7°C when the outer temperature dropped from 45°C to 0°C. As mentioned above, glistening-like vacuole formation can be triggered by a 3°C decrease in body temperature (37°C). Therefore, glistenings may occur in vivo if the lens experienced small aqueous humor temperature fluctuations. Shiba et al. [106] concluded that immersing AcrySof IOLs in warm water (37°C or 60°C) for a short time may alter the IOL's features. On the contrary, when the lens is left for 6 months in 15°C water, glistening formation is not observed.

Miyata and Yaguchi [107] correlated the degree of glistenings in two different hydrophobic acrylic IOLs, AcrySof MA60BM (Alcon) and Sensar AR40 (AMO), with their equilibrium water content at 30°C, 40°C, and 50°C. The 2 IOLs were also subjected to 3 temperature changes: from 37°C to 35°C, from 39°C to 35°C, and from 41°C to 35°C. IOLs were incubated in a physiological saline solution at the higher temperature for 2 hours and at the lower temperature for 30 days before being assessed for glistening formation. The equilibrium water content was higher in the Sensar IOL than in the AcrySof IOL, but the temperature-related change of the equilibrium water content was greater for the AcrySof IOL. It is in those IOLs whose water content varies significantly with temperature (i.e., temperature-dependent) where phase separation (water and glistenings) occurs. When temperature changed from 37°C to 35°C, glistening formation was not observed in either IOLs, but when it changed from 39°C to 35°C, glistenings were observed in AcrySof IOL, and when it went down from 41°C to 35°C, they were observed in both IOLs.

Glistenings can be evaluated subjectively by means of a slit lamp and by slit lamp photography of the IOL at high magnification. The size and number of glistenings can be quantified by either manual or digital image analysis. Miyata et al. [105] classify glistening into the following grades: 0 = no glistenings; 1 = up to 50/mm^3^, 2 = up to 100/mm^3^, and 3 = up to 200/mm^3^. Some authors also suggested quantifying glistenings employing light scattering. Klos et al. [108] proposed the use of Scheimpflug camera photography for glistening evaluation, as irregularities, damage, or disturbances in the transparency of the IOL material with lower light scattering. Out of the 41 AcrySof IOLs that were evaluated 1 year after implantation, glistenings were observed in all of them and it formed throughout the IOL's volume. No correlation between visual acuity, scotopic vision, or brightness acuity test and the glistening grade was found. Behndig and Monestam [109] described a method for quantifying glistenings that relied on Scheimpflug photography associated with IOL light scattering quantification using an image analysis program. Glistenings were observed in all AcrySof MA60AT or SA60AT IOLs; its grade correlated well with the length of the postoperative period. They also found that glistenings were more prominent near the IOL surfaces, especially the anterior one. Nevertheless, Mackool and Colin [110] consider that Scheimpflug photography is unsuitable for glistening quantification in IOLs, due to the fact that it has not been confirmed yet that the method is able to distinguish between glistening-related light scatter and light scatter due to other variables, such as the aqueous IOL interface, PCO, or the presence of biological materials on the IOL surface. Glistenings are usually distributed throughout the entire IOL optic [111]. Clinically observed glistenings are usually up to 10 *μ*m in diameter [112], although the size of glistenings observed during in vitro studies goes up to 20 *μ*m [113].

There are several factors influencing glistening formation, such as IOL material composition, manufacturing technique, IOL packaging, follow-up period, IOL diopter power, performance of phacotrabeculectomy, condition such as glaucoma or those that break down the BAB, and specific ocular medications (some anti-inflammatory agents). Glistenings have been observed in a variety of materials [114–118], including silicone, hydrogel, hydrophobic acrylic, and PMMA. However, most of the studies available in the literature on glistening formation describe them in association with hydrophobic acrylic IOLs [114–116]. Tognetto et al. [114] evaluated glistenings in 7 types of foldable IOLs (2 silicone, 3 hydrophilic acrylic, and 2 hydrophobic acrylic). They found varying degrees of glistenings in all IOLs, irrespective of the manufacturing material, but it was the AcrySof group that showed a higher percentage and a greater density of glistenings. Ronbeck et al. [116] assessed lens glistening effects in PMMA, silicone, and hydrophobic acrylic IOLs in a long-term study (11–13 years). The AcrySof hydrophobic acrylic IOL had a significantly higher degree of lens glistenings compared to the silicone and PMMA ones. Although it is likely that the various hydrophobic acrylic materials available on the market exhibit different behaviors in terms of glistening formation [14, 24], most of the peer-reviewed studies found in the literature focus on the AcrySof material, whereas relatively few evaluated other hydrophobic acrylic IOLs.

AcrySof packaging was also found to play an important role in the process of glistening formation. Omar et al. [103] carried out an in vitro study to compare glistening formation in AcrySof acrylic hydrophobic IOLs that relied on either the AcryPack or the Wagon Wheel packaging systems. Glistening formation occurred in both types of packaging, although those AcrySof IOLs packaged in Wagon Wheel did not develop glistenings when kept under constant body temperature. Furthermore, the IOLs in the AcryPack exhibited significantly more microvacuoles. This may be due to the large plastic case that holds the lens and the folding device [103, 111].

Moreno-Montanes et al. [119] studied the clinical factors influencing the frequency and intensity of glistenings by assessing 129 eyes of 94 patients that had undergone phacoemulsification and implantation of an AcrySof MA30BA IOL (Alcon). Glistenings occurred in 38 cases (29.5%) after 20.6 ± 11.5 months postoperatively (range: 1 to 50 months). They found a significant direct correlation between the frequency of glistenings and the following factors: more time elapsed between surgery and evaluation, higher IOL dioptric power, postoperative inflammation, and joint phacotrabeculectomy procedure. As for glistening intensity, it was directly correlated with the time elapsed after the surgery and the presence of postoperative inflammation. Glistenings developed more frequently in cases of phacotrabeculectomy but not after combined phacoemulsification and deep sclerectomy [119]. Other studies also found less glistening formation in lower dioptric power IOLs (≤20.0 D); this could be due to the fact that IOL thickness within a given model usually increases with IOL dioptric power. Therefore, glistenings may be more likely to accumulate in the thicker IOL matrix material in higher power IOLs [120, 121]. Nevertheless, recent studies did not find a significant correlation between the degree of glistenings and the IOL's dioptric power [116, 122]. As for the time evolution of this phenomenon, most studies show that glistenings increase in frequency and size with time up to approximately 3 years postoperatively [105, 117, 123]. A reasonable hypothesis is that the incidence and degree of glistenings may increase until the IOL is completely hydrated and all available voids within the polymer network are visible as glistenings as a result of temperature fluctuations [115]. Contrariwise, other authors found no significant association between glistening grade and duration of the follow-up period [120, 124].

Glistenings have been found to be associated with some conditions such as glaucoma [120] or those that break down the BAB [125], as well as concurrent medications or ophthalmic solutions. Colin et al. [120] assessed the correlation between clinical and demographic factors in 260 eyes implanted with different AcrySof IOL models. They found a potential association between the frequency of glistenings and the incidence of glaucoma. The authors hypothesized that this was due to an interaction of the material with the pathology of glaucoma or to the chronic topical medication used to lower IOP. Active ingredients or preservatives present in glaucoma medications may lead to the rupture of the BAB, thus modifying the aqueous humor composition and increasing the glistening rate [126, 127]. Schweitzer et al. [128] also found a significant association between the increase of glistenings and the number of topical glaucoma medication that the patient instilled on a daily basis.

Regarding clinical impact, most clinical studies show no association between glistening occurrence and a decrease in visual acuity (Table 2) [24, 119–122, 129, 130]. Nevertheless, there are a few reports on the possible impact upon contrast sensitivity function under specific conditions [112, 131, 132] (Table 2). Colin and Orignac [122] evaluated 97 eyes from 65 patients implanted with an AcrySof IOL at 18 ± 17 months of follow-up. They found that 40% of eyes had grade 0 glistenings, 32% had grade 1, and 28% had grade 2 glistenings. There were no statistically significant differences in visual acuity and contrast sensitivity across glistening-grade groups. They concluded that the intensity of glistenings was not associated with a reduction in visual acuity or contrast sensitivity at any spatial frequency evaluated. In a previous study, Colin et al. [120] evaluated the incidence of severe glistenings in a large series of AcrySof IOL wearers (model SN60AT, SN60WF, SA60AT, or MA) and assessed the potential correlation between glistenings and clinical (length of follow-up, IOL model, IOL power, Nd:YAG capsulotomy, visual acuity, spherical equivalent, ocular and systematic diseases, and medication) and demographic (age and gender) factors. In this retrospective evaluation of a series of 260 AcrySof IOLs which found glistenings in approximately 60% of IOLs, the results suggest a potential association between the incidence of glistenings and IOL power and the presence of glaucoma, but not between glistenings and age, gender, IOL model, length of follow-up, visual acuity reduction, or the presence of any of the most common ocular and systemic diseases and medications. Monestam and Behndig [121] followed 103 eyes implanted with the AcrySof IOL for 10 years. The patients were divided into different groups according to the degree of glistenings. They did not find any impact upon visual function or high- and low-contrast visual acuity, even in patients having severe glistening. Chang and Kugelberg [130] compared the development of glistenings after implantation of hydrophobic (AcrySof SA60AT) and hydrophilic IOLs (BL27). Nine years postoperatively, patients with a hydrophobic IOL developed more glistenings than those with the hydrophilic IOL, but glistenings did not affect visual acuity or contrast sensitivity. On the contrary, Gunenc et al. [131] found statistically significant differences in terms of contrast sensitivity at a high spatial frequency (12 cycles per degree) between eyes where glistening formation had occurred and those eyes that had not developed it. Xi et al. [132] concluded that severe glistenings may have an influence on high spatial frequency contrast sensitivity and reduce light sensitivity, but they did not find any impact on visual acuity. Christiansen et al. [124] reported that all patients (42 eyes) implanted with an AcrySof IOL showed some degree of glistenings. They found statistically significant differences in visual acuity in 24% of the eyes—those having severe glistenings (≥2+)—but there was no evidence that contrast sensitivity had been negatively affected by this glistening phenomenon.

Some authors have associated glistenings with intraocular straylight [121, 133–137]. Monestam and Behndig [121] found that most patients that had undergone surgery 10 years before had severe glistening and a high level of light scattering resulting from their IOLs but with no impact on visual acuity at high or low contrast. Hayashi et al. found more glistenings and surface scattering in those eyes implanted with acrylic IOLs versus silicone or PMMA IOLs, but these data were not significantly correlated with visual function or optical aberrations. Recent studies also reported the presence of straylight in eyes implanted with hydrophobic IOLs resulting from subsurface nanoglistenings, but these findings did not lead to visual acuity deterioration [135–137]. In contrast, Colin and Orignac [122] did not show any correlation between glistenings and intraocular light scatter.

Glistenings has decreased noticeably over the last years. A recent study by Thomes and Callaghan [138] reported significantly reduced levels of glistenings, measured in vitro, in newer AcrySof IOL models as a result of the continuous improvements implemented since 2003 in the manufacturing process, including manufacturing equipment, environmental controls, and tightened process controls/specifications. They found an 87% decrease in mean microvacuole density for the AcrySof IOLs manufactured in 2012 versus those produced in 2003. Packer et al. also showed the safety and effectiveness in a glistening-free hydrophobic acrylic IOL (enVista IOL). In a prospective series of 122 subjects, the authors showed no glistenings of any grade for any subject after 6 months of follow-up [139].

## 5. Conclusions

There is clear evidence that the IOL biomaterial is one of the main factors influencing PCO, ACO, and glistening formation. Most of the studies showed lower PCO rates with hydrophobic than with hydrophilic and PMMA IOL materials due to the effects outlined by Linnola in the “Sandwich theory” [15]. However, there are more controversial findings when comparing hydrophobic and silicone IOL materials: in this setting, there are additional factors that have an influence on the outcome of the cataract procedure. This is due to the fact that these two materials have both optimal properties to prevent PCO formation and, consequently, secondary factors come into play, such as edge design. Indeed, some authors consider the optic edge as the main factor preventing PCO. It has been shown that sharp edges lead to lower PCO formation than round ones, thanks to the barrier that is created, which hinders LEC migration. Nevertheless, not all square edges are the same. Edge sharpness can vary across IOL models, in the same manner that IOL material composition can vary across IOL designs. PCO and Nd:YAG laser capsulotomy rates are influenced by both IOL biomaterials and optic edge design, the best outcomes being observed for hydrophobic or silicone material IOLs having sharp edges. Other factors, such as haptics design, optic size, and the presence of aspheric surfaces, could also have a minor influence on PCO formation.

As for anterior capsule opacification, most authors have reported that ACO rates are lower for hydrophobic than for silicone and, specifically, for hydrophilic IOL materials. Other factors, such as thin optics, plate-haptics and single-piece IOLs, are additional risk factors for capsule contraction.

Glistening formation seems to be directly related with the IOL material and its composition. Most of the studies showed higher levels of glistening formation with hydrophobic IOLs, than with other materials. Nonetheless, most clinical studies showed no correlation between glistening formation and impaired visual performance.

## Figures and Tables

**Table 1:** Posterior capsule opacification (PCO) and neodymium:YAG (Nd:YAG) rates of several scientific articles published between 2000 and 2014.

Authors	IOL model	IOL characteristics	Eyes (*n*)	Study design	Follow-up	PCO rates (%)	*p* value	Nd:YAG rates	*p* value
Kucuksumer et al. [29]	Acrysof MA60BM (Alcon)	3-piece, hydrophobic acrylic	32	Contralateral & randomized study	1 year	93.7% grade 0 6.3% grade 1	<0.001	0%	—
	MemoryLens	3-piece, hydrophilic acrylic	32			40.6% grade 0 28.1 grade 1 25% grade 2 6.3% grade 3		0%	
	Acrysof MA60BM (Alcon)	3-piece, hydrophobic acrylic	21	Contralateral & randomized study	3 years	80.9% grade 0 14.4% grade 1 4.7% grade 2	<0.05	0%	0.46
	MemoryLens	3-piece, hydrophilic acrylic	21			4.7% grade 0 52.4% grade 1 19% grade 2 9.5% grade 3 14.4% grade 4		19%	
Oner et al. [33]	Acrysof MA30BA (Alcon)	3-piece, rectangular-edge hydrophobic acrylic	80	Consecutive series	16–22 months	8.70%	<0.05	14.30%	—
	Opsia-Agena 550	Round-edge, PMMA	77			24.70%		26.30%	
Hayashi et al. [39]	MZ60BD (Alcon)	Single-piece, PMMA	90	Prospective & randomized study	Up to 2 years	—	<0.0001	28.90%	—
	SI-30NB (AMO)	Silicone	83			—		14.40%	
	Acrysof MA60BM (Alcon)	3-piece, hydrophobic acrylic	96			—		4.20%	
Schauersberger et al. [44]	Acrysof (Alcon)	3-piece, square-edge, hydrophobic acrylic	25	Prospective study	3 years	85% grade 0 15% grade 1	0.323	—	—
	CeeOn 911A (AMO)	3-piece, square-edge, silicone	25			95% grade 0 5% grade 1		—	
Abhilakh Missier et al. [19]	AcrySof MA30BA/MA60BM (Alcon)	3-piece, hydrophobic acrylic	107	Retrospective & contralateral study	3 years	19.6% grade 0 53.3% grade 1 16.8% grade 2 10.3% grade 3	<0.0001	2.8%	<0.05
	AA4203VF (Staar)	Plate haptic, silicone	107			6.5% grade 0 26.2% grade 1 22.4% grade 2 44.9% grade 3		23.1%	
Bender et al. [21]	812A/Storz P497UV (Pharmacia)	PMMA	63	Retrospective study	6 months	21.9%/27.2%	<0.0001	—	—
	AcrySof MA30/SA31 (Alcon)	3-piece/1-piece, hydrophobic acrylic	33			10.5%/6.2%			
	SI-31 (AMO)	Silicone	22			15.30%			
	Hydroview H61 (B&L)	Hydrophilic acrylic	22			65.00%			
	812A/Storz P497UV (Pharmacia)	1-piece, PMMA	63	Retrospective study	2 years	32.9%/45.5%		—	—
	AcrySof MA30/SA31 (Alcon)	3-piece/1-piece, hydrophobic acrylic	33			17.8%/18.4%			
	SI-31 (AMO)	Silicone	22			17.80%			
	Hydroview H61 (B&L)	Hydrophilic acrylic	22			62.20%			
Prosdocimo et al. [42]	CeeOn Edge (AMO)	3-piece, sharp-edge, silicone	40	Prospective study	18 months	10% without VA lost	0.0336	0%	—
	Acrysof (Alcon)	3-piece, square sharp-edge, hydrophobic acrylic	38			29% without VA lost 3% with VA lost		3%	
Auffarth et al. [20]	—	Hydrophilic acrylic	294	Retrospective study	3 years	37%	<0.001	31.10%	<0.001
	—	PMMA	384			28.30%		19.30%	
	—	Hydrophobic acrylic	421			8.90%		7.10%	
	—	Silicone	426			21.60%		16.20%	
K. Hayashi and H. Hayashi [26]	Hydroview H60M (B&L)	3-piece, hydrophilic acrylic	95	Prospective randomized contralateral study	24 months	—	—	28%	<0.0001
	Acrysof MA60BM (Alcon)	3-piece, hydrophobic acrylic	95			—		2%	
Heatley et al. [27]	Centerflex 570H (Rayner)	Single-piece, square-edge, hydrophilic acrylic	53	Prospective randomized contralateral study	1 year	50.30%	<0.001	2.60%	—
	AcrySof SA60AT (Alcon)	Single-piece, square-edge, hydrophobic acrylic	53			4.90%		0%	
Kugelberg et al. [30]	BL27 (B&L)	Single-piece, square-edge, hydrophilic acrylic	60	Prospective randomized study	1 year	18.20%	<0.001	3.50%	>0.05
	AcrySof SA60AT (Alcon)	Single-piece, square-edge, hydrophobic acrylic	60			4.65%		6.80%	
Hancox et al. [13]	AcrySof SN60AT (Alcon)	1-piece, hydrophobic acrylic	36	Prospective randomized contralateral study	24 months	8.83%	<0.0001	—	—
	AF-1 YA-60BB (Hoya)	Hydrophobic acrylic	36			32.44%			
Hayashi et al. [40]	Acrysof MA60AC (Alcon)	3-piece, round optic, hydrophobic acrylic	45	Prospective randomized contralateral study	12 months	—	0.5004	2.20%	>0.999
	AR40e (AMO)	3-piece, round optic, hydrophobic acrylic	45			—		2.20%	
Kohnen et al. [41]	CeeOn Edge 911A (AMO) versus Acrysof MA60BM (Alcon)	Sharp-edge, silicone versus sharp-edge, hydrophobic acrylic	139	Prospective randomized contralateral study	37 months	—	—	2.1% versus 2.1%	
	CeeOn Edge 911A (AMO) versus PhacoFlex SI40NB	Sharp-edge, silicone versus round optic edge, silicone	108			—		5.7% versus 17%	
Kugelberg et al. [31]	BL27 (B&L)	Hydrophlic acrylic	60	Prospective randomized study	2 years	—	—	42%	<0.001
	AcrySof SA60AT (Alcon)	Hydrophobic acrylic	60			—		10%	
Boureau et al. [23]	AcrySof SA60AT (Alcon)	1-piece, sharp-edge, hydrophobic acrylic	250	Restrospective study	2.9 years	13.60%	<0.001	12%	<0.001
	AR40e (AMO)	3-piece, sharp-edge, acrylic hydrophobic	254			26.80%		25.20%	
	XL-Stabi (Zeiss)	1-piece, sharp-edge, acrylic hydrophilic	263			52.90%		51%	
Ronbeck et al. [43]	809C (Pharmacia)	Round-edge, PMMA	54	Prospective randomized study	5 years	100%		54%	<0.05
	SI-40NB (AMO)	Round-edge, silicone	48			12%		29%	
	Acrysof MA60BM (Alcon)	Sharp-edge, acrylic hydrophobic	50			18%		8%	
Vock et al. [46]	Acrysof MA60BM (Alcon)	3-piece, sharp-edge, hydrophobic acrylic	98	Retrospective study	10 years	91% grade 0 4% grade 1 4% grade 2 1% grade 3	0.000073	42%	0.007
	SI-30NB/SI-40NB (AMO)	3-piece, round optic edge, silicone	44			61% grade 0 30% grade 1 9% grade 2 0% grade 3		18%	
Vock et al. [45]	CeeOn 911A (AMO)	3-piece, sharp-edge, silicone	22	Randomized and contralateral study	6 years	—	0.0016	9%	0.01
	Acrysof MA60BM (Alcon)	3-piece, sharp-edge, hydrophobic acrylic	22			—		27.20%	
Gauthier et al. [25]	AcrySof ReSTOR (Alcon)	Hydrophobic acrylic	160	Retrospective bilateral study	2 years	—	—	8.80%	0.0001
	Acri.Lisa (Zeiss)	Hydrophilic acrylic with hydrophobic surface	152			—		37.20%	
Iwase et al. [28]	AcrySof SA60AT	1-piece, sharp-edge, hydrophobic acrylic	63	Prospective randomized and contralateral study	2 years	—	<0.001	2%	<0.01
	Meridian HP60M (B&L)	1-piece, double square-edge, hydrophilic acrylic	63			—		13%	
Vasavada et al. [34]	Acrysof IQ SN60WF (Alcon) versus C-flex 570C (Rayner)	Hydrophobic acrylic versus hydrophilic acrylic	66	Prospective randomized and contralateral study	3 years	—	0	0% versus 12.9%	0.04
	Acrysof IQ SN60WF (Alcon) versus Akreos Adapt (B&L)	Hydrophobic acrylic versus hydrophilic acrylic	62			—	0	0% versus 16.1%	0.02
Zemaitiene and Jasinskas [47]	Acrysof MA30BA (Alcon)	3-piece, hydrophobic acrylic	31	Prospective randomized study	3 years	—	0.995	9%	NS
	Acrysof SA30AL (Alcon)	1-piece, hydrophobic acrylic	31			—		3.10%	
	CeeOn 911A (AMO)	3-piece, silicone	30			—		0%	
Chang et al. [24]	Acrysof SA60AT (Alcon)	1-piece, hydrophobic acrylic	40	Prospective randomized study	5–7 years	—	0.5535	22%	>0.05
	Sensar AR40e (AMO)	3-piece, hydrophobic acrylic	40			—		10%	
Leydolt et al. [14]	iMics1 NY-60 (Hoya)	1-piece, sharp-edge optimized, hydrophobic acrylic	100	Prospective, randomized and contralateral study	3 years	—	<0.001	35.60%	0.001
	Acrysof SN60WF (Alcon)	1-piece, sharp-edge, hydrophobic acrylic	100			—		13.70%	
Morgan-Warren and Smith [32]	Hoya FY60AD (HOYA)	3-piece, round-edge, hydrophobic acrylic	315	Retrospective comparative study	2 years	—	—	8.90%	0.03
	Hoya PY60AD (HOYA)	3-piece, sharp-edge, hydrophobic acrylic	254			—		4.30%	
	Acrysof SN60WF (Alcon)	1-piece, sharp hydrophobic acrylic	696			—		1.40%	0.007
Bourdiol Ducasse et al. [22]	Acrysof SN60WF (Alcon)	1-piece, square-edge, hydrophobic acrylic	126	Retrospective study	2-3 years	—	—	10.30%	
	Akreos AO-MI60 (B&L)	1-piece, square-edge, hydrophilic acrylic	89			—		36%	
	Hoya YA-60BB (Hoya)	3-piece, square-edge, hydrophobic acrylic	85			—		24.90%	
Cullin et al. [37]	AcrySof SN60 (Alcon)	Hydrophobic acrylic	375	Retrospective study	41.5 months	—	—	7.50%	<0.001
	Akreos Adapt (B&L)	Hydrophilic acrylic	350			—		17.70%	
	Tecnis Acryl Z9003 (AMO)	Hydrophobic acrylic	801			—		3.70%	
Fong et al. [38]	AcrySof SA60AT (Alcon)	1-piece, square-edge, hydrophobic acrylic	1016	Prospective cohort study	3 years	34.40%	—	—	—
	MA50BM (Alcon)	3-piece, square-edge, hydrophobic acrylic	67			50.80%		—	
	Sensar AR40e (AMO)	3-piece, round-edge, hydrophobic acrylic	156			38.50%		—	
	Akreos Adapt (B&L)/Quatrix (Croma)	Square-edge, hydrophilic acrylic	101			64.40%		—	
Ronbeck and Kugelberg [12]	809C (Pharmacia)	Round-edge, HSM PMMA	61	Prospective randomized study	12 years	—	—	57.40%	
	SI-40NB (AMO)	round-edge, silicone	59			—		28.80%	
	Acrysof MA60BM (Alcon)	Sharp-edge, hydrophobic acrylic	59			—		32.20%	

**Table 2:** Impact of glistenings on visual function with several models of AcrySof intraocular lenses (IOLs).

Author	IOL model	Eyes (*n*)	Follow-up	Results
Moreno-Montanes et al. [119]	AcrySof MA30BA	129	20 ± 11 months	No impact on visual acuity
Colin et al. [120]	AcrySof SN60AT, SN60WF, SA60AT, or MA	260	0–86 months	No impact on visual acuity
Colin et al. [129]	AcrySof SN60WF	111	±24 months	No impact on visual acuity
Monestam and Behndig [121]	AcrySof MA60BM	103	>10 years	No impact on visual acuity at high and low contrast
Colin and Orignac [122]	Several AcrySof	97	18 months	No impact on visual acuity or contrast sensitivity
Hayashi et al. [133]	AcrySof versus SI30NB versus PMMA	35	>10 years	No impact on visual acuity and aberrometry
Chang et al. [24]	AcrySof SA60AT versus Sensar AR40e	80	5–7 years	No impact on visual acuity or contrast sensitivity
Chang and Kugelberg [130]	AcrySof SA60AT & BL27	120	9 years	No impact on visual acuity or contrast sensitivity
Waite et al. [112]	AcrySof SA & SN60	20	36 months	No impact on visual acuity, aberrometry, and contrast sensitivity but at high spatial frequencies
Gunenc et al. [131]	AcrySof MA30BA & MA60BA	91	7–24 months	Decreased contrast sensitivity at high spatial frequencies
Xi et al. [132]	AcrySof SA60AT	120	2 years	No impact on visual acuity and contrast sensitivity but at high spatial frequencies
Christiansen et al. [124]	AcrySof MA30BA & MA60BM	42	6–46 months	Decreased visual acuity but not glare and contrast sensitivity

## Additional Points

*Precis*. Although cataract surgery is one of the most common surgeries performed everyday worldwide and technology and products are constantly improving day after day, we can still find undesirable effects following cataract surgery in some patients.
